# Real-world effectiveness and safety of tolvaptan in liver cirrhosis patients with hepatic edema: results from a post-marketing surveillance study (START study)

**DOI:** 10.1007/s00535-020-01691-x

**Published:** 2020-05-09

**Authors:** Isao Sakaida, Shuji Terai, Masayuki Kurosaki, Mitsuru Okada, Takahiro Hirano, Yasuhiko Fukuta

**Affiliations:** 1 Department of Gastroenterology and Hepatology, Graduate School of Medicine, Yamaguchi University, 1-1-1, Minamikoogushi, Ube City, Yamaguchi 755-8505 Japan; 2 Division of Gastroenterology and Hepatology, Graduate School of Medical and Dental Sciences, Niigata University, Niigata, Japan; 3 Department of Gastroenterology and Hepatology, Japanese Red Cross Musashino Hospital, Tokyo, Japan; 4 Department of Pharmacovigilance, Otsuka Pharmaceutical Co., Ltd., Tokyo, Japan; 5 Department of Medical Affairs, Otsuka Pharmaceutical Co., Ltd., Tokyo, Japan

**Keywords:** Ascites, Cirrhosis, Edema, Fibrosis, Tolvaptan

## Abstract

**Background:**

This large-scale post-marketing surveillance study (START study) evaluated the effectiveness and safety of tolvaptan in Japanese liver cirrhosis patients with hepatic edema in real-world clinical settings. Here, we present the final analysis outcomes.

**Methods:**

A prospective, multicenter, non-interventional study involving patients who received tolvaptan for the treatment of liver cirrhosis with hepatic edema with an insufficient response to conventional diuretics. The observation period was up to 6 months. Effectiveness evaluation included changes in body weight and clinical symptoms. Safety analysis included evaluation of adverse drug reactions (ADRs).

**Results:**

Case reports of 1111 patients were collected. Of these, 1109 were included in the safety analysis and 1098 in the effectiveness analysis. The mean age was 69.4 ± 11.5 years and 695 (62.7%) patients were male. After tolvaptan treatment, a decrease in body weight from baseline was − 2.6 ± 2.7 kg on day 7 and − 3.8 ± 4.1 kg on day 14. Moreover, clinical symptoms significantly improved over the 14-day treatment. Frequently reported ADRs were thirst (6.6%), hepatic encephalopathy (2.3%), dehydration (1.5%), and hypernatremia (1.2%). A serum sodium level of ≥ 150 mEq/L was reported in five patients (0.5%). Multivariate analyses showed that the baseline blood urea nitrogen (BUN) level (cut-off value: 22.4 mg/dL) was the predictive factor for tolvaptan treatment response.

**Conclusions:**

The results suggest that tolvaptan was effective and well-tolerated in liver cirrhosis patients with hepatic edema. In the real-world clinical setting, tolvaptan provides a useful option for the treatment of hepatic edema.

**Electronic supplementary material:**

The online version of this article (10.1007/s00535-020-01691-x) contains supplementary material, which is available to authorized users.

## Introduction

Liver cirrhosis culminates into ascites, edema, or pleural effusion in approximately half of the patients within 10 years of diagnosis [1], and is associated with significant morbidity, mortality, and healthcare expenditures [2]. Persistent hepatic edema triggers a range of subjective and objective symptoms, thereby compromising the quality of life (QOL) [3]. Therefore, improvement of edema is an important therapeutic strategy for treating these patients. Spironolactone, an aldosterone antagonist, either alone or in combination with the loop diuretic (e.g., furosemide) is considered as the first-line treatment for management of patients with liver cirrhosis and persistent edema [4]. However, some patients are diuretic-resistant and might not respond adequately to this therapy [5], e.g., those with hypoalbuminemia show a poor response with furosemide [6]. Furthermore, randomly increasing the diuretic dose is restricted, owing to the risk of adverse drug reactions (ADRs), such as worsening of renal function, activation of the rennin–angiotensin and sympathetic nervous systems, electrolyte disturbances, and hepatic coma [7]. Therefore, there is an emergent need for an effective therapeutic option over and above the conventional diuretics for the management of liver cirrhosis patients with hepatic edema.

Tolvaptan (SAMSCA^®^, JINARC^®^, JYNARQUE^®^), a selective vasopressin V_2_-receptor antagonist, has been approved in over 40 countries for the treatment of clinically significant hypervolemic and euvolemic hyponatremia including patients with heart failure, the syndrome of inappropriate antidiuretic hormone, and autosomal dominant polycystic kidney disease [8–10]. Moreover, in Japan, tolvaptan has been approved for the treatment of fluid retention in patients with heart failure or liver cirrhosis, regardless of their sodium levels, for whom conventional diuretics are not effective, and autosomal dominant polycystic kidney disease [11]. In addition, the Evidence-based Clinical Practice Guidelines for Liver Cirrhosis 2015 recommends tolvaptan as evidence level A for the treatment of ascites and impaired water excretion [12].

In various clinical trials, tolvaptan has been found to be well-tolerated and efficacious in the treatment of Japanese patients with liver cirrhosis and edema [13–15]. However, these findings were limited by the duration of the tolvaptan treatment (only for 14 days). Furthermore, the generalizability of the results is limited by the clinical trial’s strict inclusion and exclusion eligibility criteria. Real-world data were therefore required to update the risk–benefit profile of tolvaptan for Japanese liver cirrhosis patients with hepatic edema.

Here, we present the final analysis results of a large-scale, real-world post-marketing surveillance (PMS) study—START (Samsca posT-mARkeTing surveillance of tolvaptan in liver cirrhosis). The interim results of this study were published in 2017 [16]. The primary objective of this study was to analyze and confirm the efficacy and safety of tolvaptan in Japanese liver cirrhosis patients with hepatic edema.

## Methods

### Study design

The START study was a prospective, multicenter, non-interventional, real-world PMS study, which aimed to evaluate the safety and effectiveness of tolvaptan in liver cirrhosis patients with hepatic edema. The study, conducted from June 2014 to December 2017 in 233 sites across Japan was in compliance with Good Post-marketing Study Practice, an ordinance issued by the Japanese Ministry of Health, Labor and Welfare establishing the standards for implementation of PMS for all new drugs approved in Japan.

The data collection was anonymous in nature. Considering the non-interventional nature of this study, obtaining informed consent from patients and approval by the institutional review board of investigational sites were not mandatory; however, these were obtained according to the regulations of the respective investigational site. This approach was compliant with Japanese regulations for PMS studies.

### Study population

A total of 1111 patients receiving tolvaptan for the treatment of liver cirrhosis with hepatic edema with an insufficient response to conventional diuretics were included in this study. In line with the label claim, patients with anuria, those having difficulties with water intake, and hypernatremia, and pregnant women were excluded from the study. The standard observation period was specified as 2 weeks, however, it could be extended to up to 6 months or longer at the discretion of the attending physician.

### Study assessments

Demographic data prior to tolvaptan treatment, body weight, cumulative 24-h urine volume, paracentesis, symptoms related to fluid retention, and clinical manifestations accompanied by ascites (bloated feeling, lower-limb edema, loss of appetite, malaise, a feeling of pressure in the supine position, pleural effusion, and dyspnea) were recorded. Symptoms were recorded from baseline (before tolvaptan treatment) through the consecutive treatment periods. Ascites was rated according to the symptomatic grading (five points) based on the physician’s discretion: none, mild, moderate, severe, and tense. Similarly, lower limb edema and pleural effusion were rated on four points based on the physician’s discretion: none, mild, moderate, and severe. Bloated feeling, loss of appetite, malaise, a feeling of pressure in the supine position, and dyspnea were evaluated according to their presence or absence.

Improvement in hepatic edema was measured by the reduction in body weight as a marker for the decrease in ascites volume [17]. Hence, in this study, change in body weight was assessed to evaluate the effectiveness of tolvaptan treatment. Variables including age, serum creatinine, BUN, spironolactone, hepatitis B, and alcohol hepatitis were studied to identify predictors of the pharmacological action of tolvaptan. Safety was assessed by evaluating the incidence and type of adverse events (AEs) throughout the study period, and by monitoring the changes in laboratory values and vital signs at predefined time points.

### Statistical analyses

Change in body weight from baseline (before tolvaptan treatment) and clinical symptoms were analyzed in all patients, excluding those who had undergone paracentesis during tolvaptan treatment. Patients with baseline values and at least one post-baseline value were included in the analysis.

Concerning symptoms related to edema and disappearance or improvement of each symptom vs. baseline (before tolvaptan treatment) were summarized for symptomatic patients. Disappearance rates were calculated for bloated feeling, loss of appetite, malaise, a feeling of pressure in the supine position, and dyspnea if the symptoms disappeared within 14 days of tolvaptan treatment. Improvement rates were calculated for lower limb edema, pleural effusion, and ascites if the symptoms improved at least by one grade within 14 days of tolvaptan treatment.

In the responder analysis, patients were categorized as responders or non-responders. Responders were defined as those whose body weight reduced by ≥ 1.5 kg within 7 days of initiating tolvaptan treatment [18], and the rest were categorized as non-responders. To identify predictive factors associated with response to tolvaptan, univariate and multivariate logistic regression analyses were performed.

The relationship between responder rate and level of BUN was investigated using a receiver operating characteristic (ROC) analysis, using a responder as an objective variable. Subsequently, the cut-off value was calculated.

For the safety analysis, AEs for which causal relationships with tolvaptan could not be ruled out were tabulated as ADRs, using the Medical Dictionary for Regulatory Activities (MedDRA Version 20.1).

Data were expressed as a mean ± standard deviation (SD) or as proportions (%). Considering the properties of each data set, Fisher’s exact test, Chi-square test, or Student’s *t* test was used for comparing patient parameters. The Cochran–Armitage test was used for trend analysis of the dose and responder rates of tolvaptan.

Depending on the nature of data, statistical significance was defined as the *P* value < 0.05, < 0.01, or < 0.001. All statistical analyses were performed using SAS version 9.3 (SAS Institute Inc., Cary, NC, USA).

## Results

### Patient disposition and demographics

As of December 2016, case reports of a total of 1111 patients were collected. Of these, 1109 patients were included in the safety analysis and 1098 were included in the effectiveness analysis. Patients enrolled prior to the study start date were excluded.

A summary of the demographics of all patients enrolled in this study compared with those in a Phase III study [14] is presented in Table 1. The mean age of the patients was 69.4 ± 11.5 years, and the majority (62.7%) were male. The most common underlying disease among patients with liver cirrhosis was hepatitis C (42.7%), followed by alcoholic hepatitis (31.8%). The majority of cirrhotic patients belonged to Child–Pugh Grade C (51.0%). The mean baseline level of 24-h urine output was 1242 ± 685 mL. Complications of hepatocellular carcinoma (HCC) and gastroesophageal varices were observed in 43.6% and 56.7% of patients, respectively.

**Table 1:** Characteristics of patients: START study vs. phase III trial

Characteristics	START study (*N* = 1109)	Phase III trial^d^ (*N* = 162)
Age (years), mean ± SD	69.4 ± 11.5	66.3 ± 9.4
Sex, male (%)	62.7	63.4
Cause of liver cirrhosis (%)		
Hepatitis A	0.0	0.0
Hepatitis B	6.5	6.1
Hepatitis C	42.7	58.5
Alcoholic liver disease	31.8	32.9
Unidentified viral cirrhosis	0.6	12.2
Drug-induced liver disease	0.2	NR
Child–Pugh score (%)		
A (5–6 points)	3.7	0.0
B (7–9 points)	42.5	53.7
C (10–15 points)	51.0	46.3
24-h urine volume (mL), mean ± SD	1242 ± 685	1006 ± 763
Gastroesophageal varices (%)	56.7	82.9
Hepatic encephalopathy (%)	11.8	NR^a^
I	7.7	NR
II	3.2	0.0
III	0.7	0.0
Unknown	0.3	0.0
Hepatocellular carcinoma (%)	43.6	28.0
Loop diuretics^b^ (mg), mean ± SD	32.3 ± 35.9	64.5 ± 40.4
Spironolactone^c^ (mg), mean ± SD	51.8 ± 86.7	55.2 ± 57.8
Loss of body weight on day 7 (kg), mean ± SD	− 2.6 ± 2.7	− 2.0 ± 1.8

*NR* not reported, *SD* standard deviation

^a^Grade 2 or higher were excluded in Phase III trial

^b^Loop diuretics: furosemide equivalent

^c^Spironolactone: androgen deprivation therapy equivalent

^d^[14]

### Dosage and treatment period

**Fig. 1:**
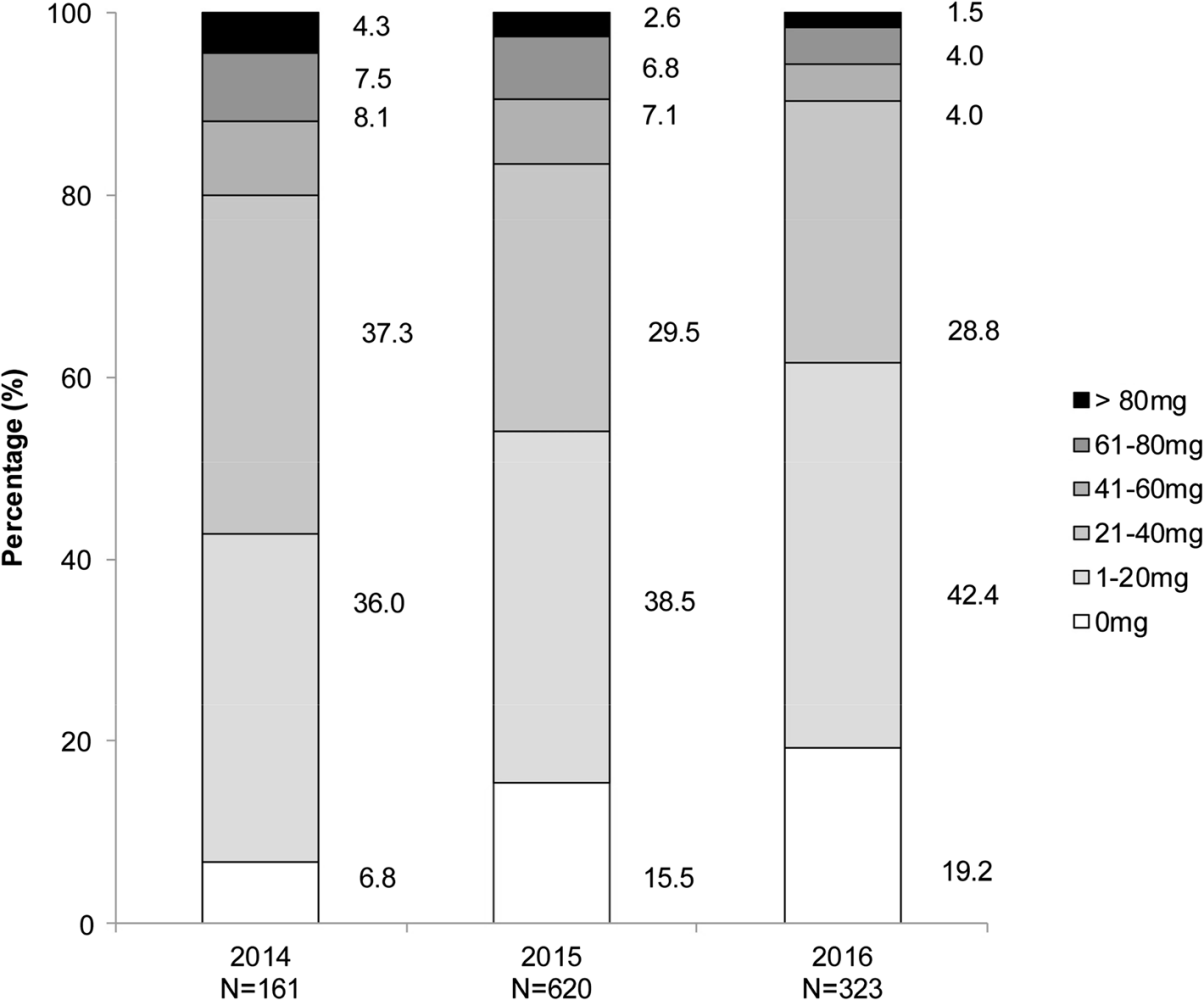
Dose of loop diuretic at the initiation of tolvaptan by year (2014–2016). The figure shows the dose of loop diuretic at different doses for years 2014–2016. The Cochran–Armitage test (*P* < 0.0001) was used for trend analysis of the dose (≤ 20 mg) of loop diuretics

Overall, 55.6% of patients received the initial daily dose as 3.75 mg and 44.4% of patients as 7.5 mg. The initial daily dose of 3.75 mg was increased to 7.5 mg within 30 days in 33.0% of the patients. Of the patients who initiated a daily dose of 7.5 mg, the dosage was maintained in 95.5% of the patients and was reduced to 3.75 mg in 3.7% of the patients. The mean initial dose of tolvaptan administered was 5.4 ± 1.9 mg. The mean daily dose of tolvaptan administered was 6.0 ± 1.8 mg. The mean duration of the treatment period was 82.0 ± 99.0 days (median 36 days; maximum 773 days). The average baseline dose of a loop diuretic and spironolactone was 32.3 ± 35.9 mg (median 20 mg/day) and 51.8 ± 86.7 mg (median 25 mg/day), respectively. In 2016, at baseline, the distribution of loop diuretics used were 0 mg (19.2%), ≤ 20 mg (42.4%), < 20–40 mg (28.8%), and ≥ 41 mg (9.6%) (Fig. 1).

### Effectiveness

**Fig. 2:**
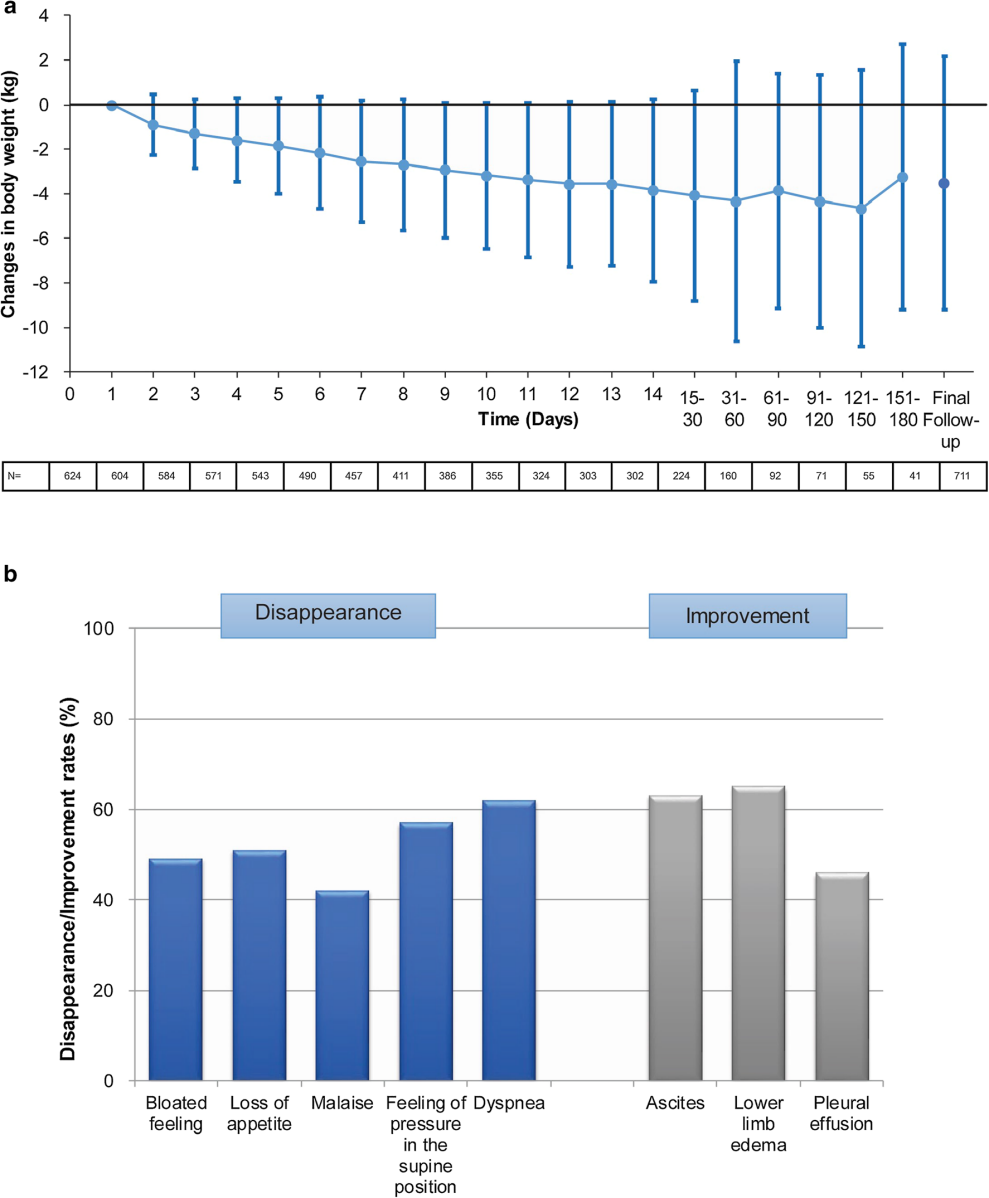
Changes in body weight (**a**) and disappearance/improvement rates in clinical symptoms (**b**) from pretreatment levels. Mean (SD) changes in body weight during the course of the study are provided. Disappearance/improvement rates are provided and ascites, lower limb edema and pleural effusion were rated based on the physician’s discretion

A significant decrease (*P* < 0.001) in body weight from baseline (before tolvaptan treatment) to Day 7 and Day 14 was reported. Tolvaptan treatment led to a reduction in body weight during the 14-day treatment period. The body weight decreased by 2.6 ± 2.7 kg on Day 7 and 3.8 ± 4.1 kg on Day 14 (Fig. 2a). Compared with pretreatment levels, the mean reductions in weight at Day 60 (*N* = 160) and Day 180 (*N* = 41) were 4.3 ± 6.3 kg and 3.2 ± 6.0 kg, respectively. In the last observation period of tolvaptan treatment, there was a mean reduction of 3.5 ± 5.3 kg in weight vs. the pretreatment values. The proportion of patients with a decrease in body weight over 1.5 kg was 64.9% on Day 7 and 71.9% on Day 14 and that over 3.0 kg was 38.0% on Day 7 and 54.6% Day 14 (Supplementary Fig. 1).

Table 2 shows the changes in body weight stratified by subgroups (HCC, albumin level, and serum sodium level). There was no significant change in body weight from baseline (before tolvaptan treatment) between patients with and without HCC (*P* = 0.2248), with albumin levels of < 2.5 g/dL and ≥ 2.5 g/dL (*P* = 0.3372), and serum sodium levels of < 125 mEq/L and ≥ 125 mEq/L (*P* = 0.2222).

**Table 2:** Change in body weight in patients treated with tolvaptan for liver cirrhosis and edema, stratified by subgroups

	*N*	Mean ± SD	Median	*P* value^a^
Hepatocellular carcinoma				
Yes	288	− 3.21 ± 4.93	− 2.70	0.2248
No	413	− 3.70 ± 5.53	− 3.20	
Albumin (g/dL)				
< 2.5	209	− 3.85 ± 5.47	− 3.40	0.3372
≥ 2.5	365	− 3.42 ± 5.11	− 2.80	
Serum Na (mEq/L)				
< 125	13	− 5.25 ± 6.86	− 3.40	0.2222
≥ 125	684	− 3.45 ± 5.25	− 2.90	

*SD* standard deviation

^a^Student’s *t* test

^*^< 0.05

**< 0.01

***< 0.001

Figure 2b represents disappearance or improvement rates in symptoms related to fluid retention within 14 days of tolvaptan treatment. The rates of disappearance of bloated feeling, loss of appetite, malaise, a feeling of pressure in the supine position, and dyspnea were 49% (353/720), 51% (165/323), 42% (186/448), 57% (204/359), and 62% (80/130), respectively. The rates of improvement in lower limb edema, pleural effusion, and ascites were 65%, 46%, and 63%, respectively. Supplementary Fig. 2 shows the changes in the proportion of patients with fluid retention and clinical symptoms from the pretreatment levels. The signs and symptoms were improved reflecting the decrease in body weight.

### Safety

The list of ADRs with the incidence rate of ≥ 0.5% is presented in Table 3. These events were reported as ADRs based on the physicians’ judgment. Thirst was the most frequently reported ADR (73 [6.6%] patients). Other frequently (> 1% patients) reported ADRs were hepatic encephalopathy (HE), dehydration, hypernatremia, and renal impairment. The majority of the ADRs occurred within 30 days of tolvaptan treatment. Hypernatremia was reported in 13 (1.2%) patients; majority of the events (46%) were reported within 3 days of tolvaptan treatment. None of the hypernatremia events were considered serious, and all were of mild or moderate intensity. None of the patients had osmotic demyelination syndrome or drug-induced liver injury, which were suspected by attending physicians. In total, 157 deaths were reported, of which 15 could not be denied to relate to tolvaptan based on the physicians’ judgement. The breakdown of these deaths is as follows. Hepatic failure, hepatic cirrhosis, hepatocellular carcinoma, and renal impairment in two patients each, and hepatic failure in combination with HE, hemothorax, gastrointestinal hemorrhage, hepatic failure accompanied by peritonitis bacterial, ascites, hepatorenal syndrome, and increased blood urea in one patient each.

**Table 3:** Incidence rate of adverse drug reactions by the timing of occurrence reported by physicians (≥ 0.5%)

Preferred term, *n* (%)	Total (*N* = 1109)	1–3 days (*N* = 1109)	4–7 days (*N* = 1088)	8–14 days (*N* = 992)	15–30 days (*N* = 782)	> 31 days (*N* = 588)	Unknown
Thirst	73 (6.6)	53 (4.8)	15 (1.4)	1 (0.1)	1 (0.1)	2 (0.3)	1
Hepatic encephalopathy	25 (2.3)	2 (0.2)	5 (0.5)	3 (0.3)	5 (0.6)	10 (1.7)	0
Dehydration	17 (1.5)	2 (0.2)	4 (0.4)	3 (0.3)	4 (0.5)	4 (0.7)	0
Hypernatremia	13 (1.2)	6 (0.5)	4 (0.4)	3 (0.3)	0	0	0
Renal impairment	11 (1.0)	3 (0.3)	1 (0.1)	4 (0.4)	1 (0.1)	2 (0.3)	0
Hyperkaliemia	9 (0.8)	1 (0.1)	4 (0.4)	2 (0.2)	0	2 (0.3)	0
Blood urea increased	8 (0.7)	0	3 (0.3)	4 (0.4)	0	1 (0.2)	0
Blood creatinine increased	7 (0.6)	0	1 (0.1)	3 (0.3)	0	3 (0.5)	0

In liver cirrhosis patients with hepatic edema, a mild increase in mean serum sodium level was reported (Supplementary Fig. 3). A serum sodium level of ≥ 150 mEq/L was reported in five patients (0.5%). An increase in serum sodium level by ≥ 10 mEq/L within 1 day was reported in four (0.4%) patients in whom the maximum increase was from 121 mEq/L at pretreatment level to 133 mEq/L on Day 1.

It was found that there was no difference in the incidences of ADRs (≥ 0.5% in total patients), including hypernatremia, and decrease in body weight between the 3.75 and 7.5 mg/day groups. Efficacy results (decrease in body weight) and ADRs of tolvaptan at doses of 3.75 mg/day and 7.5 mg/day (excluding patients who changed the dose of tolvaptan) are summarized in Supplementary Table 1 and Supplementary Fig. 4, respectively. The results indicated that tolvaptan has been prescribed in actual clinical practice at the appropriate dose according to the physician's judgement, and the results of this survey did not recommend either starting dose.

### Factors predicting the fraction of responders

**Fig. 3:**
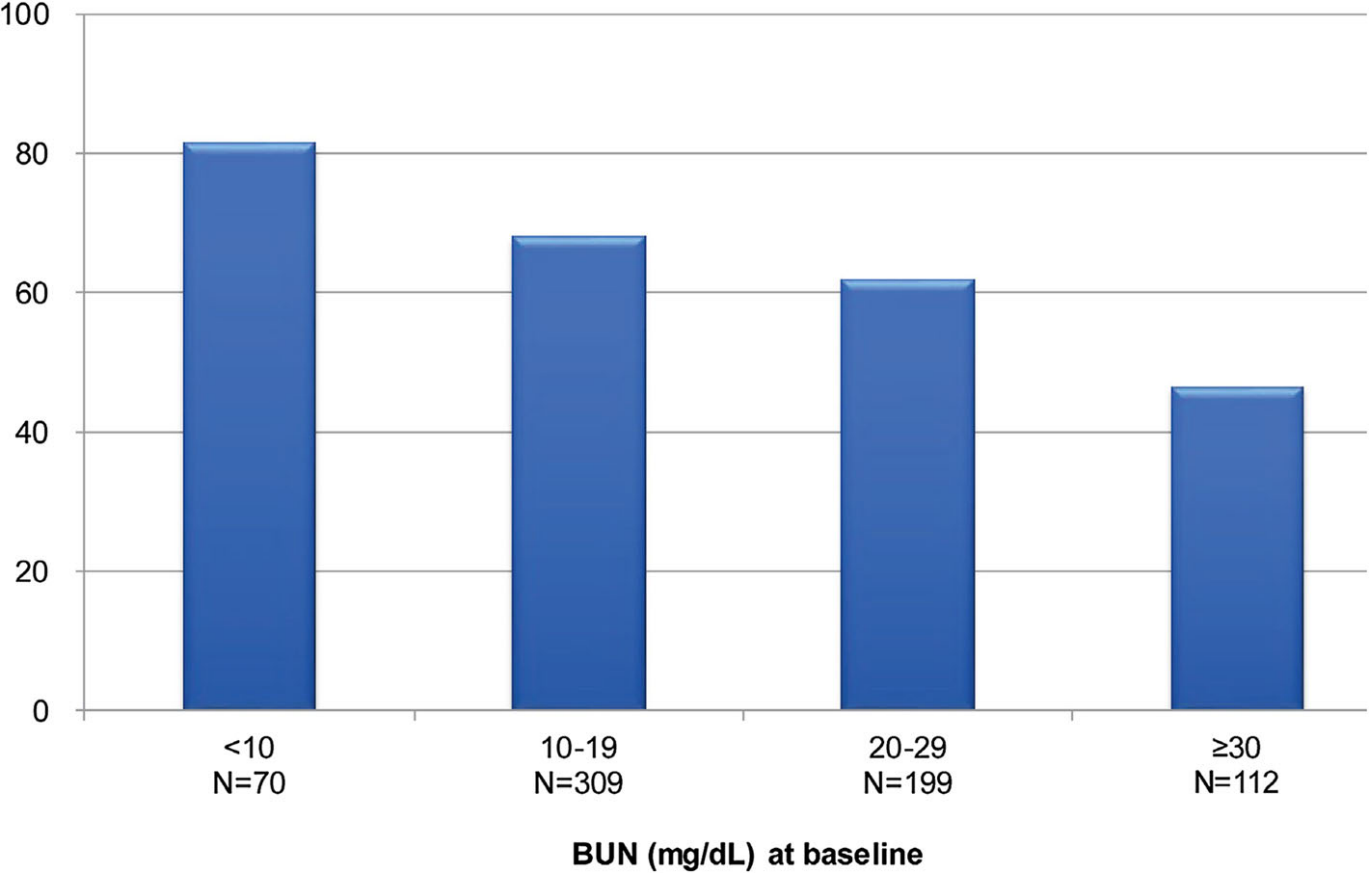
Responder rates of tolvaptan by BUN level at baseline. *BUN* blood urea nitrogen. Responders were defined as those whose body weight reduced by ≥ 1.5 kg within 1 week of initiating tolvaptan treatment. The figure shows percentage responders at different levels of BUN. Patients who had paracentesis from day 1 to 7 of administration were excluded. The Cochran–Armitage test (*P* < 0.0001) was used for trend analysis of the responder rates

Overall, more than half of the patients (62%) turned out to be responders as per the defined criteria following 1 week tolvaptan therapy. The univariate analysis revealed that age, serum creatinine level, BUN level, presence of alcoholic hepatitis, presence of hepatitis B, and higher dose of spironolactone were significantly associated with the response (*P* < 0.05) (Table 4). However, multiple regression analysis did not reveal any significant factor predicting the response except BUN level (*P* = 0.0002) (Table 4). Based on the findings from multivariate analysis, BUN level was identified as a predictive factor for tolvaptan treatment response in liver cirrhosis patients with hepatic edema. The lower levels of BUN were associated with a higher response rate (*P* < 0.0001) (Fig. 3). The cut-off value of response/no-response determined for the level of BUN by ROC analysis was 22.4 mg/dL.

**Table 4:** Responder analysis

Patients’ characteristics: responder vs. non-responder				
Characteristics	Total (*N* = 841)	Responder (*N* = 525)	Non-responder (*N* = 316)	*P* value
Age (years)	69.3 ± 11.4	68.5 ± 11.2	70.7 ± 11.6	0.0088**
Sex, male (%)	62.2	61.3	63.6	0.5570
Serum albumin (g/dL), mean ± SD	2.6 ± 0.5	2.6 ± 0.5	2.6 ± 0.5	0.3232
Serum creatinine (mg/dL), mean ± SD	1.0 ± 0.5	1.0 ± 0.5	1.1 ± 0.5	0.0013**
Blood urea nitrogen (mg/dL), mean ± SD	21.3 ± 11.8	19.4 ± 10.2	24.6 ± 13.7	< 0.0001***
Dear alcoholic hepatitis (%)	31.8	35.1	26.3	0.0093**
Hepatitis B (%)	6.5	5.1	8.9	0.0432*
Hepatitis C (%)	42.9	41.5	45.3	0.3141
Hepatocellular carcinoma (%)	42.7	40.4	46.5	0.0966
Loop diuretics^a^ (mg), mean ± SD	36.9 ± 24.0	37.5 ± 22.5	35.8 ± 26.3	0.3471
Spironolactone^b^ (mg), mean ± SD	65.1 ± 88.1	70.7 ± 100.2	54.6 ± 26.3	0.0258*
Tolvaptan (mg) mean ± SD	5.4 ± 1.9	5.5 ± 1.9	5.3 ± 1.9	0.2626

Multiple regression model for predicting responder			
Parameter	Odds ratio	95% CI	*P* value
Age	0.994	0.976–1.012	0.5051
Serum creatinine	1.586	0.900–2.795	0.1109
Blood urea nitrogen	0.954	0.931–0.978	0.0002***
spironolactone	1.002	0.999–1.005	0.1327
Hepatitis B	0.537	0.276–1.048	0.0686
Alcoholic hepatitis	1.193	0.772–1.845	0.4263

Multiple regression model for predicting responder in patients with or without HCC			
Parameter	Odds ratio	95% CI	*P* value
Patients with HCC			
Age	0.998	0.965–1.033	0.9174
Serum creatinine	1.568	0.661–3.723	0.3078
Blood urea nitrogen	0.941	0.905–0.978	0.0023**
Spironolactone	1.001	0.997–1.005	0.5354
Hepatitis B	0.348	0.139–0.871	0.0241*
Patients without HCC			
Age	0.985	0.964–1.006	0.1603
Serum creatinine	1.733	0.763–3.934	0.1888
Blood urea nitrogen	0.957	0.926–0.989	0.0096**
Spironolactone	1.003	0.999–1.007	0.1200

Responders: patients who lost ≥ 1.5 kg of body weight within 1 week of tolvaptan treatment

Patients who had paracentesis by day 1–7 of tolvaptan administration were excluded

*CI* confidence interval, *HCC* hepatocellular carcinoma, *SD* standard deviation

^*^< 0.05

**< 0.01

***< 0.001

^a^Loop diuretics: furosemide equivalent

^b^Spironolactone: androgen deprivation therapy equivalent

All predictive variables were analyzed in patients with or without HCC (Table 4). The lower BUN values were found to be the significant predictors for both patients with HCC and without HCC (*P* < 0.01). The cut-off values of response/no-response determined for the level of BUN by ROC analysis were 22.7 mg/dL and 24.3 mg/dL for patients with and without HCC, respectively.

## Discussion

Overall, the results of this PMS study are consistent with the previous studies and indicate that tolvaptan is effective and well-tolerated in Japanese liver cirrhosis patients with hepatic edema [13–15]. Treatment with tolvaptan led to a significant reduction in body weight and disappearance or an improvement in symptoms related to hepatic edema.

The data from the current study suggest that in real-world practice, tolvaptan is prescribed to more serious patients vs. clinical studies. Compared with a previous phase III study [14], the patients enrolled in the current study were slightly older (69.4 ± 11.5 years vs. 66.3 ± 9.4 years) and more critical as 3.9% had grade 2 HE; the previous Phase III study excluded these patients. In addition, the current study had a greater proportion of patients with complications of HCC (43.6% patients) compared with the previous phase III study (28.0%) [14].

Tolvaptan should be used in combination with other diuretics, such as loop diuretics or aldosterone antagonists [8]. Another observation in this study was that in real-world practice, lower doses of furosemide in combination with tolvaptan were administered when compared with clinical trials. The baseline dose of furosemide was 32.3 ± 35.9 mg when compared with that in the previous Phase III clinical trial of tolvaptan (64.5 ± 40.4 mg), [14]. Additionally, we investigated the dose of furosemide combined with tolvaptan by year in the present study. The dose of furosemide lowered year by year; in 2016, approximately 60% of the patients received furosemide at 20 mg or less (Fig. 1). This rationale is justified as the higher doses of conventional diuretics can cause ADRs such as renal impairment [7]. Instead, adding tolvaptan to a treatment regimen seems to be a better option than using conventional diuretics; tolvaptan does not seem to impair renal function in clinical practice [19–21], thereby reducing the ADRs in clinical practice.

Cumulative data suggest that tolvaptan reduces body weight and improves the signs of liver cirrhosis and edema [14, 18]. The present study demonstrated a sustained reduction in body weight during the 14-day tolvaptan treatment. Overall, the reduction in body weight observed in this study was higher than that reported in the Phase III study (START study: Day 7, 2.38 kg; Phase III study: Day 7, 1.95 kg) [14]. The long-term effectiveness of tolvaptan was examined in the final analysis of the START study, wherein patients were treated and followed up to 6 months. The findings of this study suggest that tolvaptan was effective in reducing hepatic edema, as observed by reduction in body weight, and this benefit sustained over a 6-month long treatment. Tolvaptan also reduced the clinical symptoms that otherwise impact a patient’s survival, functional capacity, and QOL [22]. The findings of the current study are further supported by the results from recent studies, where treatment with tolvaptan improved the prognosis in liver cirrhosis patients with ascites [23–25].

The present study demonstrated that, unlike standard diuretics, the pharmacological action of tolvaptan is independent of the baseline serum albumin or sodium levels as observed in previous studies [14, 26, 27]. Furthermore, this study showed that the reduction in body weight is independent of the presence of HCC. Overall, tolvaptan seems to have an edge while treating liver cirrhosis patients with edema since these patients often have extremely low levels of albumin and sodium in end-stage liver diseases [28]. Tolvaptan as an add-on therapy to loop diuretics can therefore be considered an optimal therapeutic option in patients with insufficient response to loop diuretics.

The present study demonstrated that tolvaptan is generally safe and well-tolerated in liver cirrhosis patients with hepatic edema, as observed previously [13, 14]. Thirst, HE, and hypernatremia were the most frequent ADRs, as expected. Thirst and hypernatremia are generally a result of the pharmacological action of tolvaptan [8]. Hypernatremia is one of the serious ADRs of tolvaptan in patients with heart failure [29]. However, the frequency of hypernatremia (> 150 mEq/L) reported in this study is lower, and none of the patients had an osmotic demyelination syndrome. This could be due to the fact that hypernatremia is more frequently reported with higher doses (15 mg) of tolvaptan [29] while patients in the current study received a lower dose (3.75–7.5 mg) of tolvaptan. Hyponatremia is common in patients with advanced cirrhosis and is associated with an increased risk of HE [28]. The incidence of HE in this analysis was 2.3%, and 1.1% of the patients had a history of HE. Sakaida et al. [14] reported that the incidence of HE in the tolvaptan group (4.9%) was equivalent to that in the placebo group (5.0%). Overall, the frequency of HE reported in patients treated with tolvaptan was considered to be lower when compared with other diuretics (23%) [7] and 16–21% in those with decompensated cirrhosis [30]. Tolvaptan has been reported to normalize the serum sodium levels in patients with hyponatremia. An improved serum sodium concentration was maintained by prolonged tolvaptan treatment with an acceptable margin of safety. This may be the reason for the differences observed between tolvaptan and other diuretics.

The data show that the baseline BUN levels were the independent predictor for the reduction in body weight (cut-off value 22.4 mg/dL), in-line with an earlier study [31]. The cut-off values of BUN were reported to be 29.0 mg/dL by Chishina et al. [32], 25.2 mg/dL by Sakaida et al. [33], 26.9 mg/dL by Kawaratani et al. [34], and 28.2 mg/dL by Atsukawa et al. [27]. BUN is considered to be an indicator of arterial underfilling and renal function [35, 36]. The patients with lower BUN levels are presumed to have enough water in the blood vessels to excrete by tolvaptan, or have better renal perfusion, or have normal kidney function. These results suggest that tolvaptan is more beneficial in patients with lower BUN levels.

The present study has some limitations, including those inherent to survey reports and the lack of an active comparator group. Furthermore, the participating centers were chosen based on the interest of the physicians from these centers, and this could have contributed to a potential bias toward favorable results. Additionally, the potential for bias due to confounding by unadjusted/unmeasured factors owing to the non-randomized, observational study design should be noted.

## Conclusion

This final analysis of the START study provides a robust real-world evidence from a large population of liver cirrhosis patients with edema. The results demonstrated that tolvaptan is effective in patients with hepatic edema and more critical patients are treated with tolvaptan in combination with lower doses of spironolactone and/or furosemide. Safety results showed that the onset of adverse drug reactions was within a range of what was expected based on the pharmacological properties and the already known safety profile of tolvaptan. The present study also suggested that the baseline level of BUN as a predictive factor for the responders treated with tolvaptan.

## Electronic supplementary material

Below is the link to the electronic supplementary material.

Supplementary file1 (DOCX 6581 kb)
