# Supplementary material for: Real-world effectiveness and safety of tolvaptan in liver cirrhosis patients with hepatic edema: results from a post-marketing surveillance study (START study)
Source: J Gastroenterol. 2020 May 9;55(8):800–10. doi: 10.1007/s00535-020-01691-x (PMC7376514; doi:10.1007/s00535-020-01691-x)
Supplement: Supplementary file 1 — Supplementary file1 (DOCX 6581 kb) [file 535_2020_1691_MOESM1_ESM.docx]

***Journal of Gastroenterology***

**SUPPLEMENTARY INFORMATION**

**Real-World Effectiveness and Safety of Tolvaptan in Liver Cirrhosis Patients with Hepatic Edema – Results from a Post-Marketing Surveillance Study (START Study)**

Isao Sakaida,^1^ Shuji Terai,^2^ Masayuki Kurosaki,^3^ Mitsuru Okada,^4^ Takahiro Hirano,^5^ Yasuhiko Fukuta^4^

^1^Department of Gastroenterology and Hepatology, Graduate School of Medicine, Yamaguchi University, Ube, Yamaguchi, Japan

^2^ Division of Gastroenterology & Hepatology, Graduate School of Medical and Dental Sciences, Niigata University, Niigata, Japan

^3^Department of Gastroenterology and Hepatology, Japanese Red Cross Musashino Hospital, Tokyo, Japan

^4^Department of Pharmacovigilance, Otsuka Pharmaceutical Co., Ltd., Tokyo, Japan

^5^Department of Medical Affairs, Otsuka Pharmaceutical Co., Ltd., Tokyo, Japan

**Correspondence**: Isao Sakaida, MD, PhD. E-mail: sakaida@yamaguchi-u.ac.jp.

**Supplementary Figures**

**Supplementary Figure 1.** Changes in the proportion of patients with decrease in body weight from pretreatment levels

The figure shows the proportion of patients with a decrease in body weight within defined ranges, during the course of the study.

**
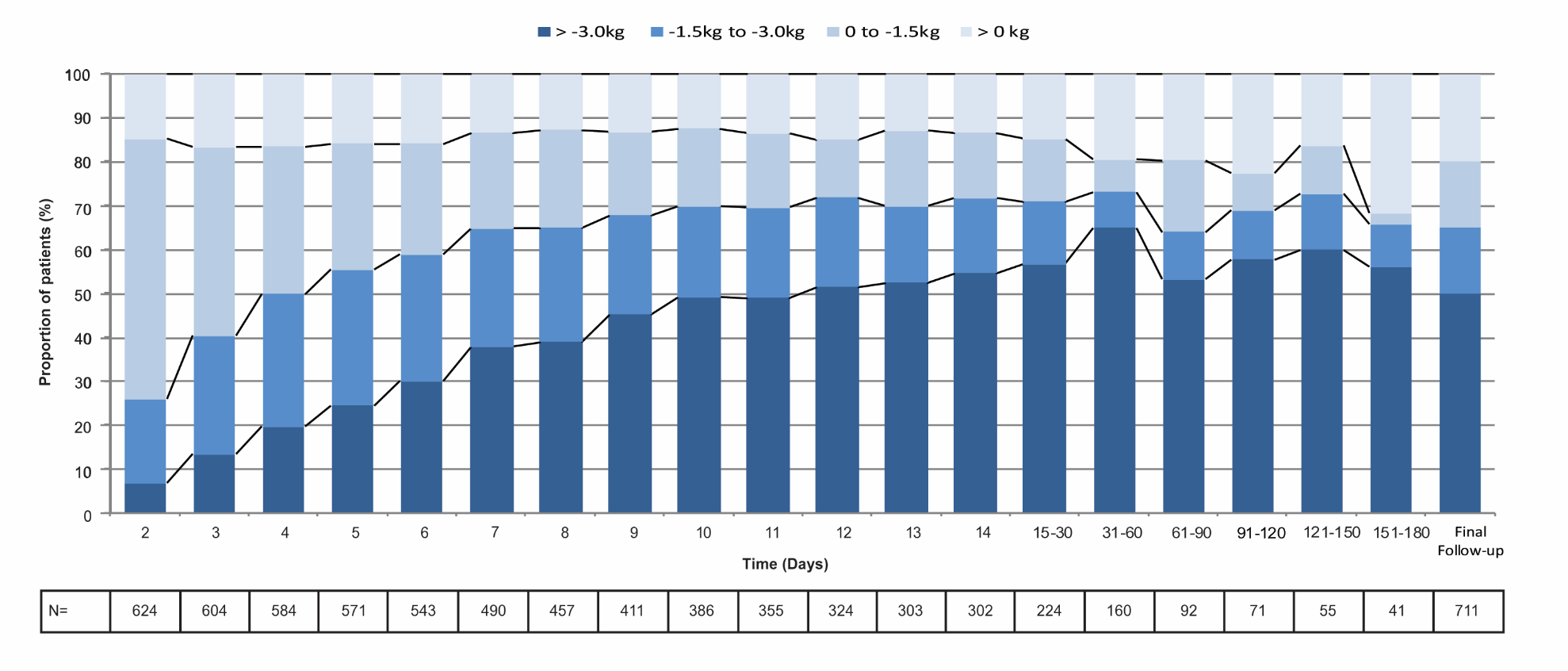
**

**Supplementary Figure 2.** Change in proportion patients with fluid retention and clinical symptoms from baseline.

The figure shows changes in the proportion of patients with fluid retention and clinical symptoms from the pretreatment levels.


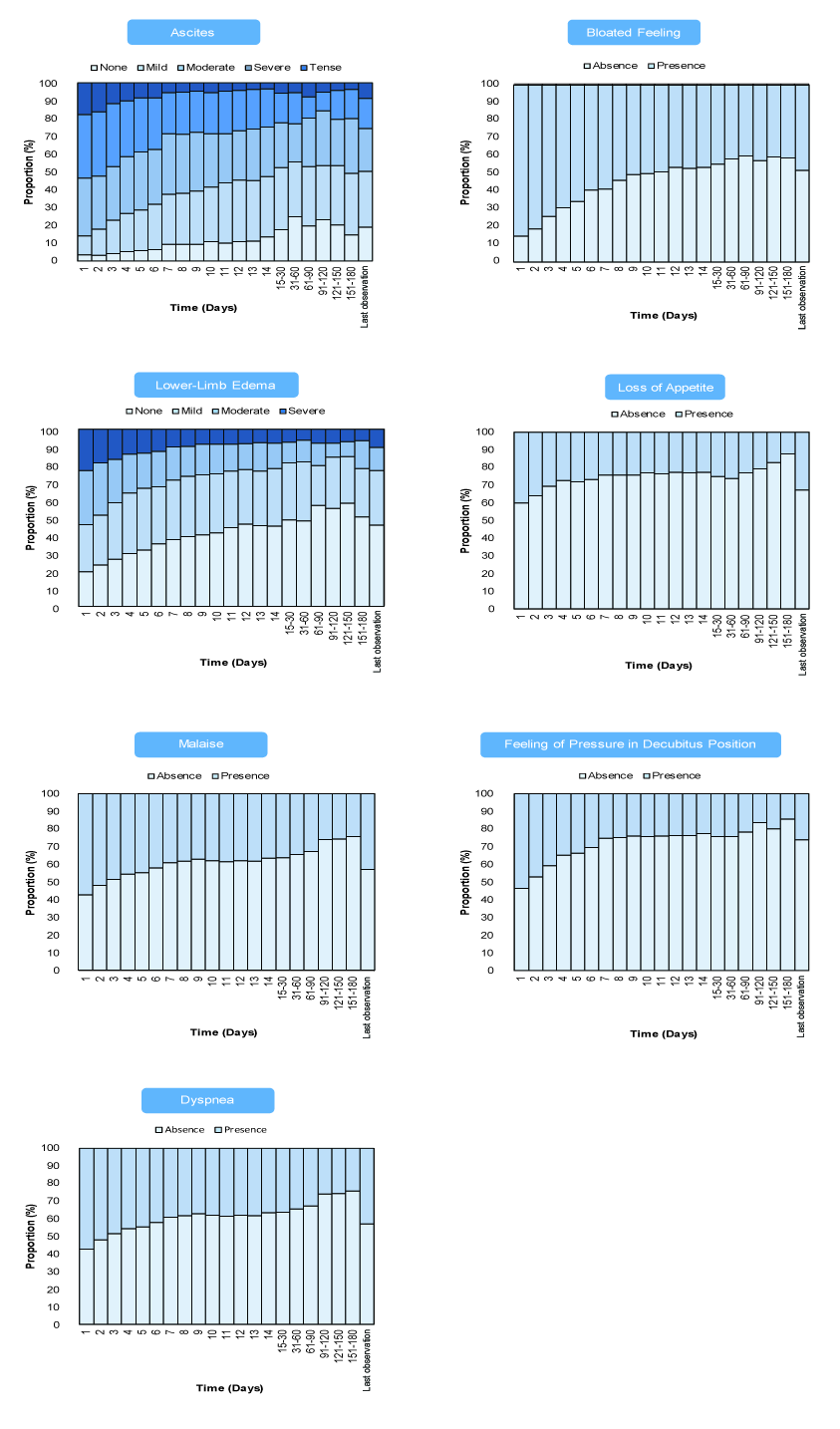


**Supplementary Figure 3.** Changes in serum sodium level from baseline

**
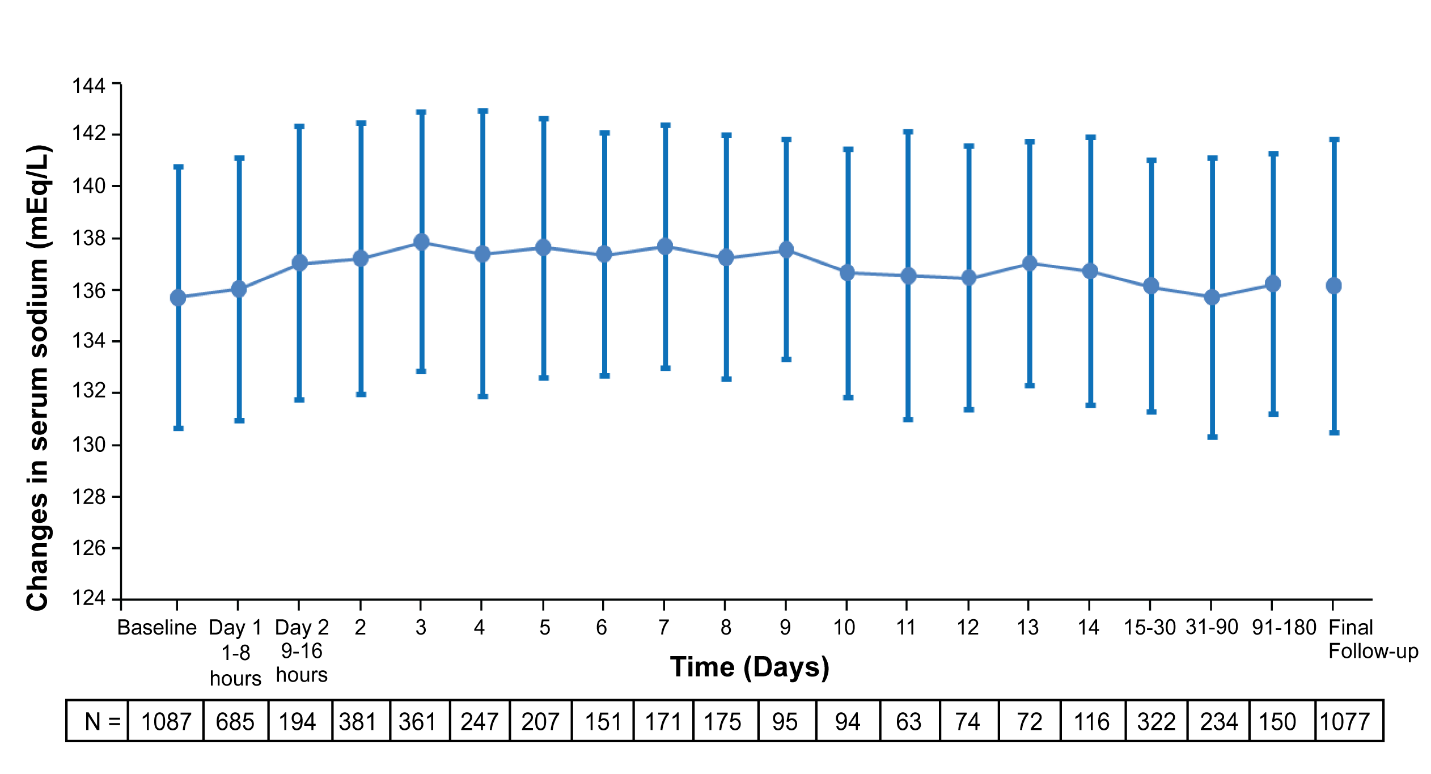
**

Data is expressed as mean±SD

**Supplementary Figure 4.** Changes in body weight from baseline according to fixed dose of tolvaptan.


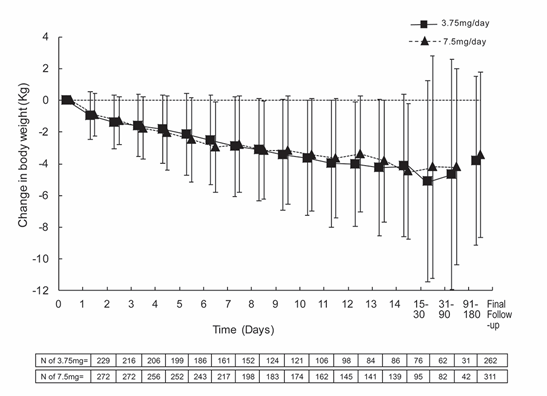


The figure showed changes in body weight at the fixed dose of tolvaptan at 3.75 mg/day and 7.5 mg/day (excluding patients who changed the dose of tolvaptan). Data is expressed as mean±SD.

**Supplementary Table 1.** Incidence of adverse drug reactions according to fixed dose of tolvaptan

| Preferred term, n (%) | | 3.75mg/day  (N=390) | | 7.5mg/day  (N=468) | |
| --- | --- | --- | --- | --- | --- |
|  |  |  |  |  |  |
|  | Thirst | 24 | (6.15) | 33 | (7.05) |
|  | Hepatic encephalopathy | 9 | (2.31) | 8 | (1.71) |
|  | Dehydration | 4 | (1.03) | 8 | (1.71) |
|  | Hypernatremia | 4 | (1.03) | 7 | (1.50) |
|  | Renal impairment | 5 | (1.28) | 3 | (0.64) |
|  | Hyperkaliemia | 3 | (0.77) | 5 | (1.07) |
|  | Blood urea increased | 2 | (0.51) | 3 | (0.64) |
|  | Blood creatinine increased | 1 | (0.26) | 1 | (0.21) |

The table showed the incidence of adverse drug reactions at the fixed dose of tolvaptan at 3.75 mg/day and 7.5 mg/day (excluding patients who changed the dose of tolvaptan)
